# ASFL-YOLOX: an adaptive spatial feature fusion and lightweight detection method for insect pests of the Papilionidae family

**DOI:** 10.3389/fpls.2023.1176300

**Published:** 2023-06-14

**Authors:** Lijia Xu, Xiaoshi Shi, Zuoliang Tang, Yong He, Ning Yang, Wei Ma, Chengyu Zheng, Huabao Chen, Taigang Zhou, Peng Huang, Zhijun Wu, Yuchao Wang, Zhiyong Zou, Zhiliang Kang, Jianwu Dai, Yongpeng Zhao

**Affiliations:** ^1^ College of Mechanical and Electrical Engineering, Sichuan Agricultural University, Ya’an, China; ^2^ College of Resources, Sichuan Agricultural University, Chengdu, China; ^3^ College of Biosystems Engineering and Food Science, Zhejiang University, Hangzhou, China; ^4^ College of Electrical and Information Engineering, Jiangsu University, Zhenjiang, China; ^5^ Institute of Urban Agriculture, Chinese Academy of Agricultural Sciences, Chengdu, China; ^6^ Regulation Department, China Telecom Corporation Limited Sichuan Branch, Chengdu, China; ^7^ College of Agronomy, Sichuan Agricultural University, Chengdu, China; ^8^ Changhong Digital Agriculture Research Institute, Sichuan Changhong Yunsu Information Technology Co., Ltd, Chengdu, China

**Keywords:** pest detection, YOLOX, GhostNet-ECA, ASFF, pruning strategy, plant protection

## Abstract

**Introduction:**

Insect pests from the family Papilionidae (IPPs) are a seasonal threat to citrus orchards, causing damage to young leaves, affecting canopy formation and fruiting. Existing pest detection models used by orchard plant protection equipment lack a balance between inference speed and accuracy.

**Methods:**

To address this issue, we propose an adaptive spatial feature fusion and lightweight detection model for IPPs, called ASFL-YOLOX. Our model includes several optimizations, such as the use of the Tanh-Softplus activation function, integration of the efficient channel attention mechanism, adoption of the adaptive spatial feature fusion module, and implementation of the soft Dlou non-maximum suppression algorithm. We also propose a structured pruning curation technique to eliminate unnecessary connections and network parameters.

**Results:**

Experimental results demonstrate that ASFL-YOLOX outperforms previous models in terms of inference speed and accuracy. Our model shows an increase in inference speed by 29 FPS compared to YOLOv7-x, a higher mAP of approximately 10% than YOLOv7-tiny, and a faster inference frame rate on embedded platforms compared to SSD300 and Faster R-CNN. We compressed the model parameters of ASFL-YOLOX by 88.97%, reducing the number of floating point operations per second from 141.90G to 30.87G while achieving an mAP higher than 95%.

**Discussion:**

Our model can accurately and quickly detect fruit tree pest stress in unstructured orchards and is suitable for transplantation to embedded systems. This can provide technical support for pest identification and localization systems for orchard plant protection equipment.

## 1 Introduction

As agricultural production continues to expand, fruit tree pests have become a critical factor limiting fruit tree yield and quality (Li et al., 2021; Xin and Wang, 2021; Ji and Wu, 2022). Insect pests, particularly those from the Papilionidae family, are a significant issue due to their high seasonal incidence, extensive damage, and potential for causing serious losses in agricultural production (Trkolu and Hanbay, 2019; Zhan et al., 2021; Toscano-Miranda et al., 2022; Yu et al., 2022). However, the existing pest identification models used by orchard plant protection equipment cannot balance inference rate and accuracy, and fail to meet the demand for pest detection in unstructured orchard environments. Therefore, it is crucial to propose an efficient, accurate, and fast pest detection method for an orchard and agricultural production.

In recent years, scholars worldwide have made significant progress in researching intelligent detection technology for plant pests and diseases (Di and Li., 2022). The most commonly used target detection algorithms are deep learning-based RCNN family (R-CNN, Fast R-CNN, and Faster R-CNN) and SSD (Single Shot MultiBox Detector) (Chen et al., 2022; Lamping et al., 2022; Xiao et al., 2022). Brahimi et al. (2017) classified nine diseases based on the AlexNet model and reduced labor costs. Srdjan et al. (2016), Mohanty et al. (2016), and Ferentinos (2018) used the Convolutional Neural Networks (CNN) model to identify diseases, achieving good recognition results for more than 50 species. Liu et al. (2017) and Ashqar et al (Ashqar and Abu-Naser, 2019). used CNN models to identify different diseases on one plant leaf, promoting plant protection efficiency. Tetila et al. (2019) first segmented images using the simple linear iterative clustering (SLIC) method and then used the CNN classification model to identify soybean leaf pests. Wang et al. (2017) studied the damage level of apple leaf diseases using CNN and obtained an accuracy of 90.4%.

The YOLO series are one-step detection algorithms. YOLO was initially proposed by Redmon et al (Redmon et al., 2016). It implements region generation and target classification directly, and divides the feature map in the form of a grid during prediction, resulting in a dramatic increase in detection speed. However, the very first YOLO algorithm has some limitations such as imprecise localization and low detection accuracy (Fu et al., 2021; Wang and Liu, 2021; Qi et al., 2022). Consequently, the YOLO series has attracted many researchers’ attention because of fast inference speed and high precision, and YOLOv4 to YOLOv7 were subsequently introduced (Liu et al., 2022; Roy and Bhaduri, 2022; Ying et al., 2022). Zha et al. (2021) proposed the YOLOv4-mf model with YOLOv4 as the base network and MobileNetv2 as the feature extraction block, which improves the detection accuracy of forest pests. However, the model has issues with high computational complexity and long processing time when handling large-scale image data. Liu et al (Liu and Wang, 2020) optimized the feature layer of the YOLOv3 model with image pyramids to achieve multi-scale feature detection and improve detection accuracy and speed. However, in practical applications, the model suffers from sensitivity to target scales and difficulty in detecting small objects. Guo et al. (2022) proposed an automatic monitoring scheme based on yellow sticky board sampling and YOLO-SIP to achieve rapid and accurate monitoring of flying pests in vegetables, avoiding traditional manual sampling. However, optimization of factors such as the number and placement of sticky traps during sampling and detection still requires further investigation.

In this study, we use YOLOX as the basic framework, introduce a lightweight feature extraction network Ghostnet to replace the backbone network, and design Tanh-Softplus (TS) to replace the original Sigmoid-Weighted Linear Units (SiLU) activation function. We combine the efficient channel attention (ECA) mechanism and adaptive spatial feature fusion (ASFF) to implement model pruning strategy and candidate frame optimization to achieve better performance.

## 2 Materials and methods

### 2.1 Materials

#### 2.1.1 Image acquisition

**Figure 1:**
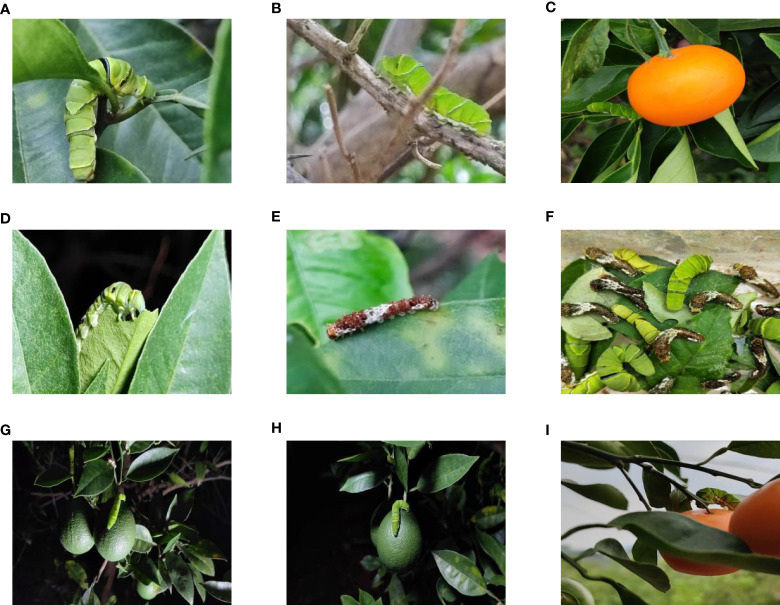
A portion of the image dataset of IPPs: **(A)** Blocked by leaf; **(B)** Blocked by branch; **(C)** Blocked by fruit; **(D)** Night photo; **(E)** Young larva; **(F)** High larvae density; **(G)** Side lighting; **(H)** Front lighting; **(I)** Backlighting.

Most IPPs host crops such as Phellodendron and Rutaceae’s citrus (Riaz et al., 2020), making a citrus orchard a representative choice for image collection. The dataset used in this study was gathered from an orchard located in Ya’an City, Sichuan Province, China. The collected data comprised images of Papilionidae pests taken during different periods (daytime, nighttime, evening, etc.), under varying lighting conditions (front lighting, backlighting, side lighting, etc.), and from different shooting angles (front angle, side angle, oblique side angle, etc.). This ensured that the types of images corresponded to the actual growth of pests in their natural environment. A total of 35,000 images with clear target contours and textures were selected, and a portion of the image dataset is displayed in **Figure 1**.

From **Figure 1**, it is apparent that the fruit trees in unstructured orchards have lush branches and leaves, with numerous pests being concealed by leaves, branches, or fruits. The background and texture details of pests differ under different lighting conditions. Additionally, the growth of Papilionidae larvae is divided into five age groups, 1-3 instars (young) and 4-5 instars (old). The phenotypic characteristics of young and old larvae differ significantly, as shown in **Table 1**. Specifically, the body surface of 1-3 instar larvae is brown and resembles bird droppings, while the body surface of 4-5 instar larvae is green, smooth, and features odoriferous glandular horns.

**Table 1:** Common Papilionidae larvae.

larva name	the backside of old larva	side view of the top-aged larva	the backside of young larva
Papilio xuthus Linnaeus	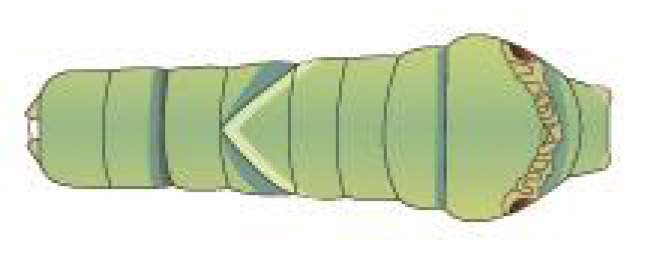	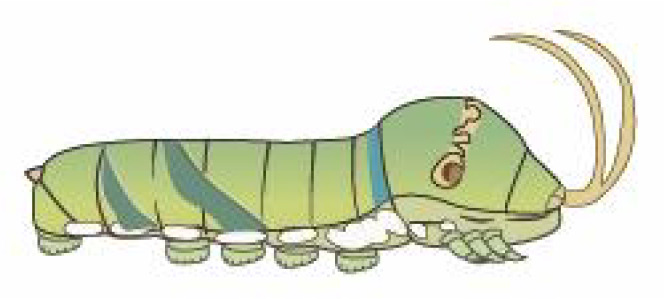	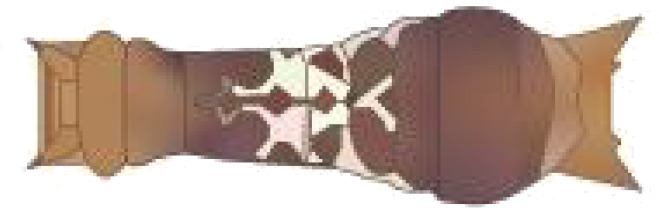
Papilio polytes Linnaeus	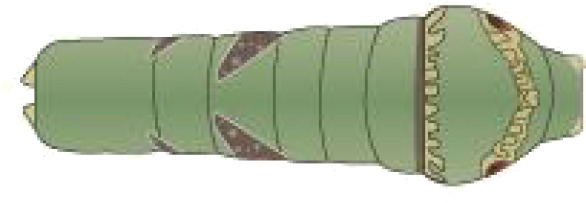	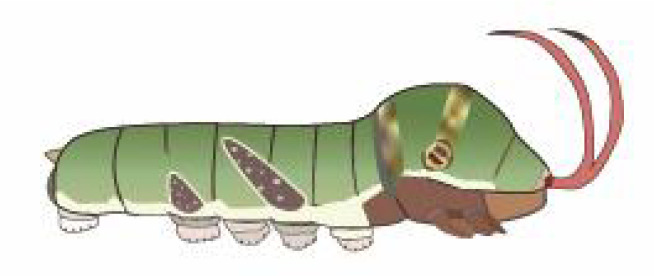	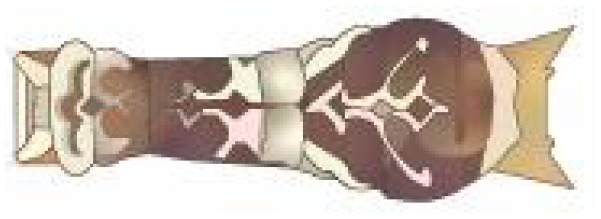
Papilio protenor amaura Jordan	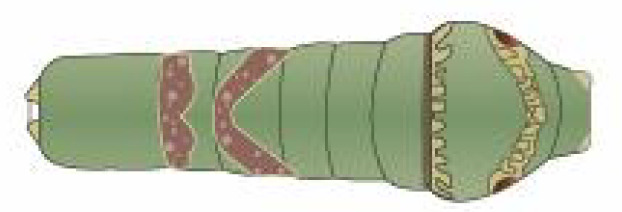	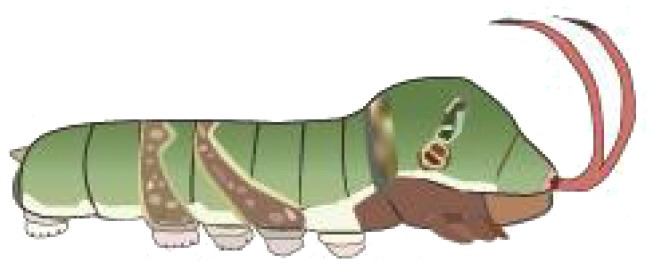	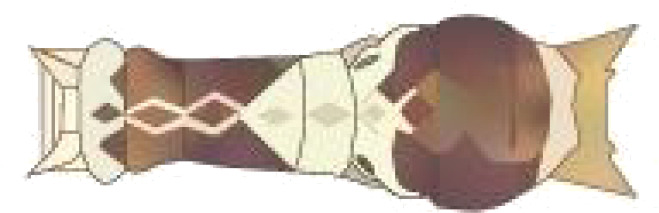
Papilio demoleus Linnaeus	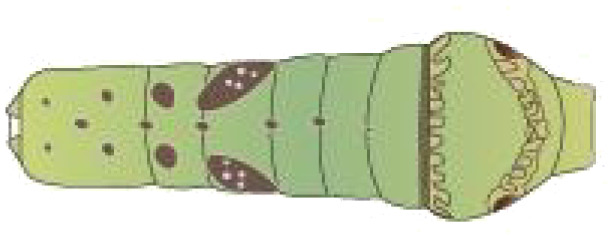	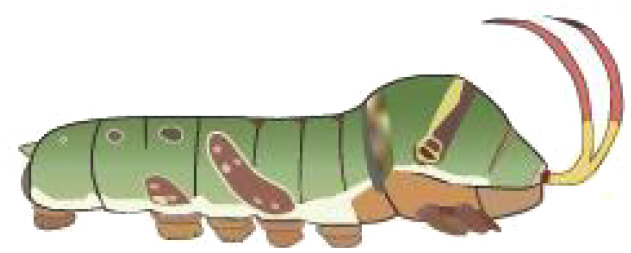	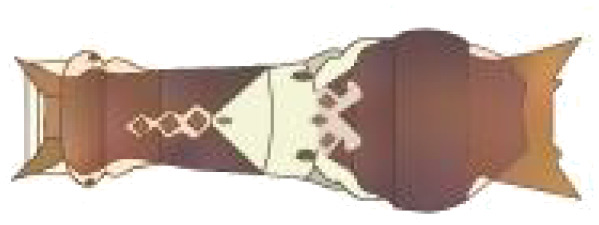

#### 2.1.2 Dataset augmentation and preparation

**Figure 2:**
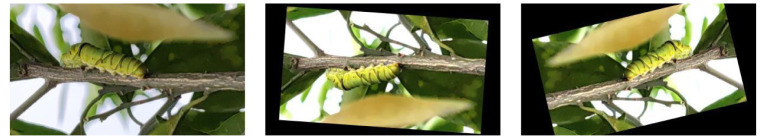
A part of the enhanced images.


Having a sufficient number of samples is a prerequisite for the successful application of deep neural networks (DNN). This study employs batch operations on images in the training set and utilizes various image processing techniques such as translation, blurring, affine, rotation, flipping, and splicing to expand the original data by five times, resulting in 175,000 images. This data augmentation enriches the dataset and enhances the generality of the detection model while avoiding overfitting. In this study, we have improved the data augmentation methods, using standard techniques to randomly adjust the contrast and brightness of the images. The image’s brightness is adjusted by adding or subtracting a certain factor to its pixel according to equation (1), while the contrast is changed by randomly multiplying the image pixels by a certain factor. The merged image can recover some of the color information to improve the feature extraction effect of the model. A portion of the enhanced images is shown in **Figure 2**.

(1) $${\tilde{x}}_{i}=x_{i}\cdot \omega +\psi$$

**Figure 3:**
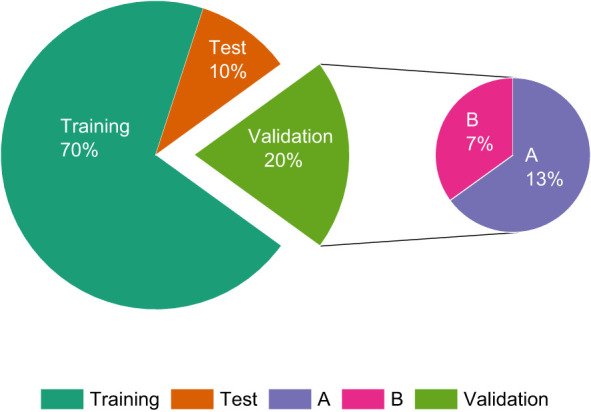
Pie chart of the distribution of IPPs dataset quantity.

We used the LabelImg tool to label the pest targets in the image, labeling the young larvae as “young” and the old larvae as “old.” The files generated by labeling are stored in the PASCAL VOC dataset format. The dataset is randomly divided into a training set, a test set, and a validation set in the ratio of 7:2:1. In the test set, samples are categorized as A if the average occlusion of the target is less than 30%, i.e., light occlusion, and as B if they have heavy occlusion. The distribution of the dataset is shown in **Table 2**; **Figure 3**.

**Table 2:** Detailed distribution of dataset quantity.

dataset		number of images
Train set		122500
Validation set		17500
Test set	A	22757
	B	12243
	A+B	35000

### 2.2 Methods

#### 2.2.1 Network structure of YOLOX

The YOLO series has been widely used in various fields due to its excellent real-time detection performance. In the YOLO series and its variants, the mainstream YOLOv3 and YOLOv5 are anchor-based target detection methods, and the prediction results are affected by the clustering results of the prior boxes. The extreme proportion of the prior box affects the detection performance of the model. YOLOX incorporates the advantages of the early YOLO series and introduces new techniques to significantly improve detection performance. A label assignment strategy is introduced to solve the problem that the detection performance is affected by the prior box. YOLOX can be divided into S, M, and L versions based on the network depth and width. Considering the detection accuracy and speed, YOLOX-x is chosen as the fundamental network for the IPPs larvae detection task in this study.

**Figure 4:**
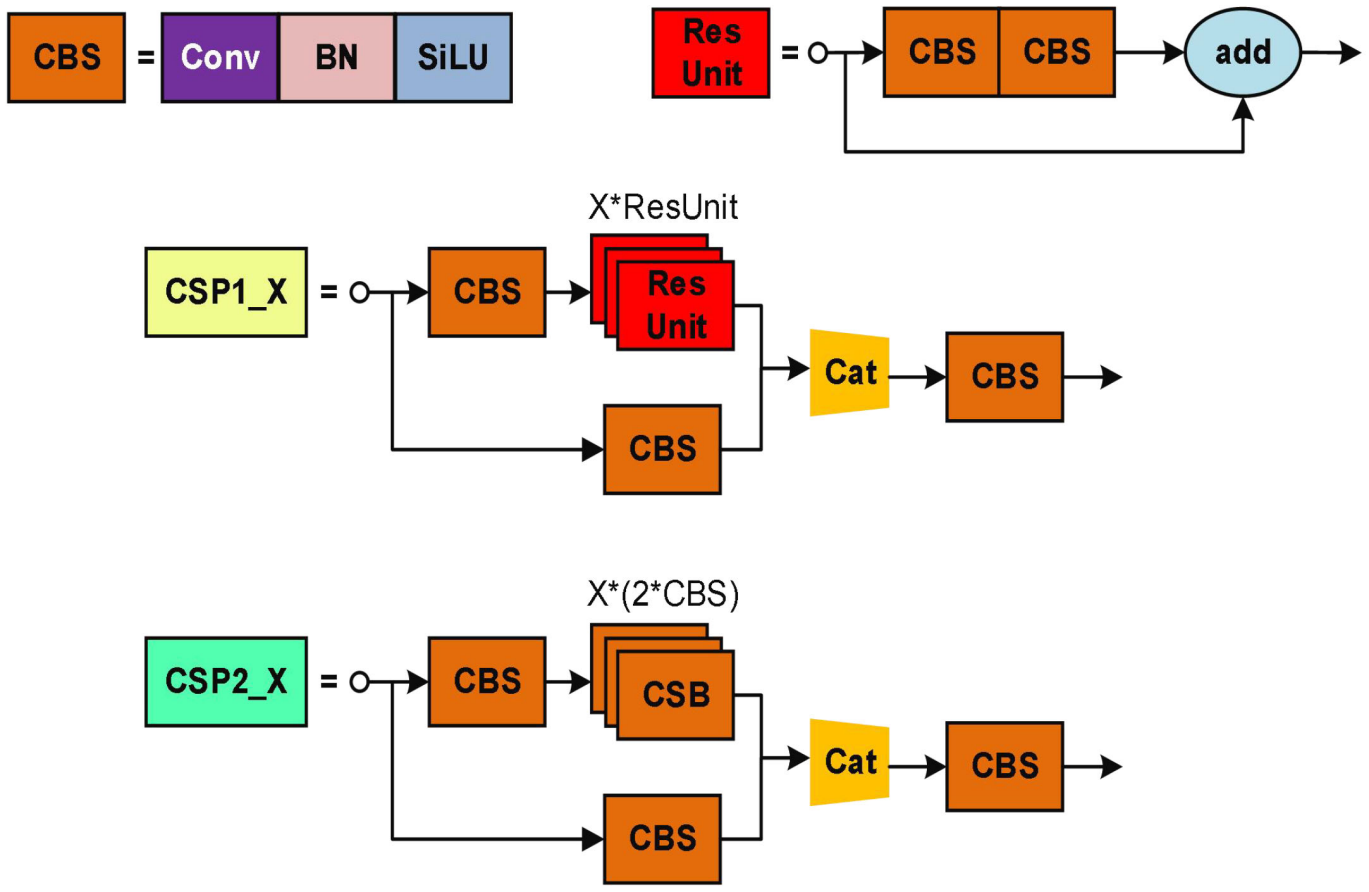
The network structure of CSP in YOLOX. (The symbol "*" represents multiplication).

The YOLOX network consists of three parts: the backbone, neck, and head (Ge et al., 2021). As shown in **Figure 4**, CBS is the basic convolution in the YOLOX network, which includes Conv, BN, and SiLU, and is mainly responsible for feature extraction. BN ensures that the output of each layer and the input data distribution of the lower layer are consistent, making the model more stable during training. The SiLU activation function gives the network the ability to change nonlinearly and abstract features hierarchically in the deep model. The CSPLayer structure builds a large residual edge while stacking the residual module, which is directly connected to the end after a small amount of processing and is mainly responsible for the feature extraction of the detection model.

#### 2.2.2 Lightweight feature extraction network

**Figure 5:**
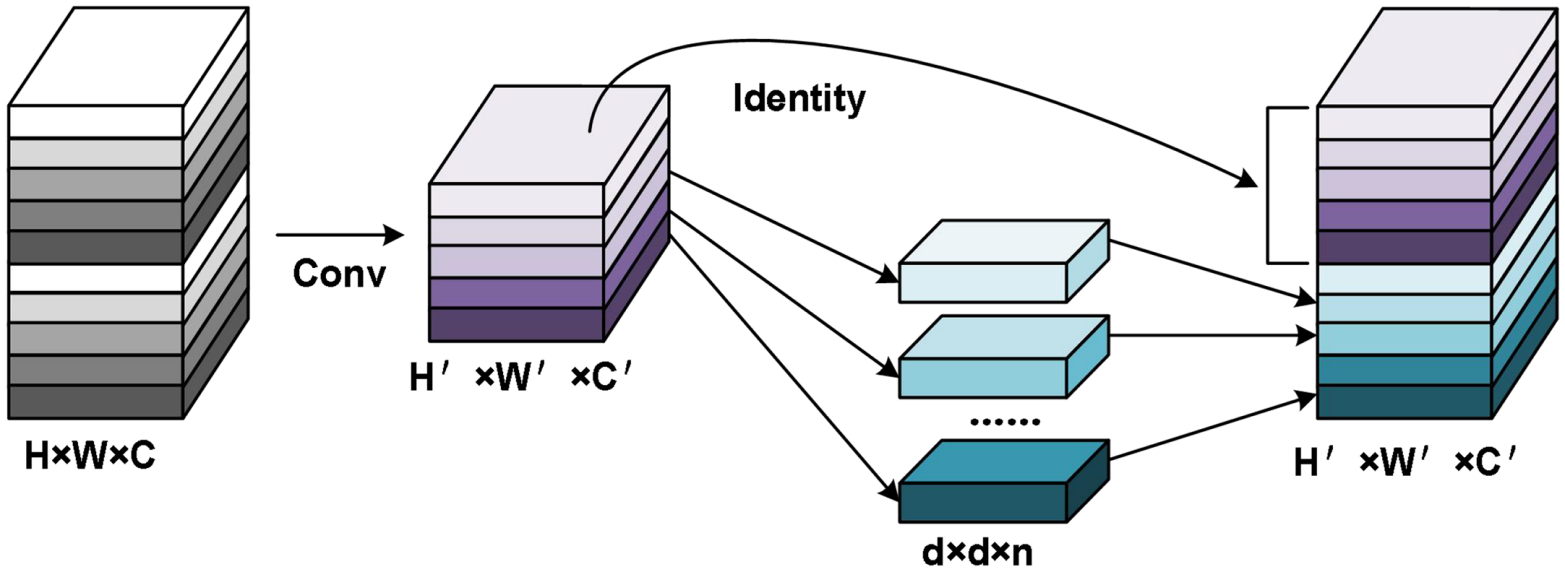
The schematic diagram of the Ghost module.

The Ghost bottleneck created by stacking Ghost modules serves as the foundation for GhostNet, a compact feature extraction network. Ghost modules can extract more information with fewer parameters than conventional convolutions. As illustrated in **Figure 5**, a Ghost module generates a real feature layer by performing standard convolution on the input feature layer, followed by a linear transformation on each channel of the real feature layer to create a Ghost feature layer. The Ghost feature layer is then combined with the real feature layer to create the full output feature layer. Assuming that the input feature map is h×w×c , the output feature map is $h^{'}\times w^{'}\times n$ , and the convolution kernel size is k×k , the input feature layer is split into s pieces. The computational cost of conventional convolution is shown in equations (2) and (3).

The Ghost bottleneck, which is created by stacking Ghost modules, forms the basis of GhostNet, a compact feature extraction network. Compared to traditional convolutions, Ghost modules can extract more information using fewer parameters. As shown in **Figure 5**, a Ghost module generates a real feature layer by performing standard convolution on the input feature layer, followed by a linear transformation on each channel of the real feature layer to create a Ghost feature layer. The Ghost feature layer is then combined with the real feature layer to create the complete output feature layer. Assuming the input feature map is h×w×c , the output feature map is $h^{'}\times w^{'}\times n$ , and the convolution kernel size is k×k , the input feature layer is divided into s pieces.

The computational cost of traditional convolution is described in formula (2), while the computational cost of the Ghost module is presented in formula (3).


(2) $$h^{'}\times w^{'}\times n\times k\times k\times c$$

(3) $$h^{'}\times w^{'}\times \frac{n}{s}\times k\times k\times c+(s-1)\times h^{'}\times w^{'}\times \frac{n}{s}\times k\times k$$

As per formula (3), the Ghost module can be considered as breaking down the multiplication operation of regular convolution into two multiplication additions. In comparison to traditional convolutions, the Ghost module achieves a model compression rate of approximately s, leading to a substantial decrease in model computation time.

#### 2.2.3 Efficient channel attention model

**Figure 6:**
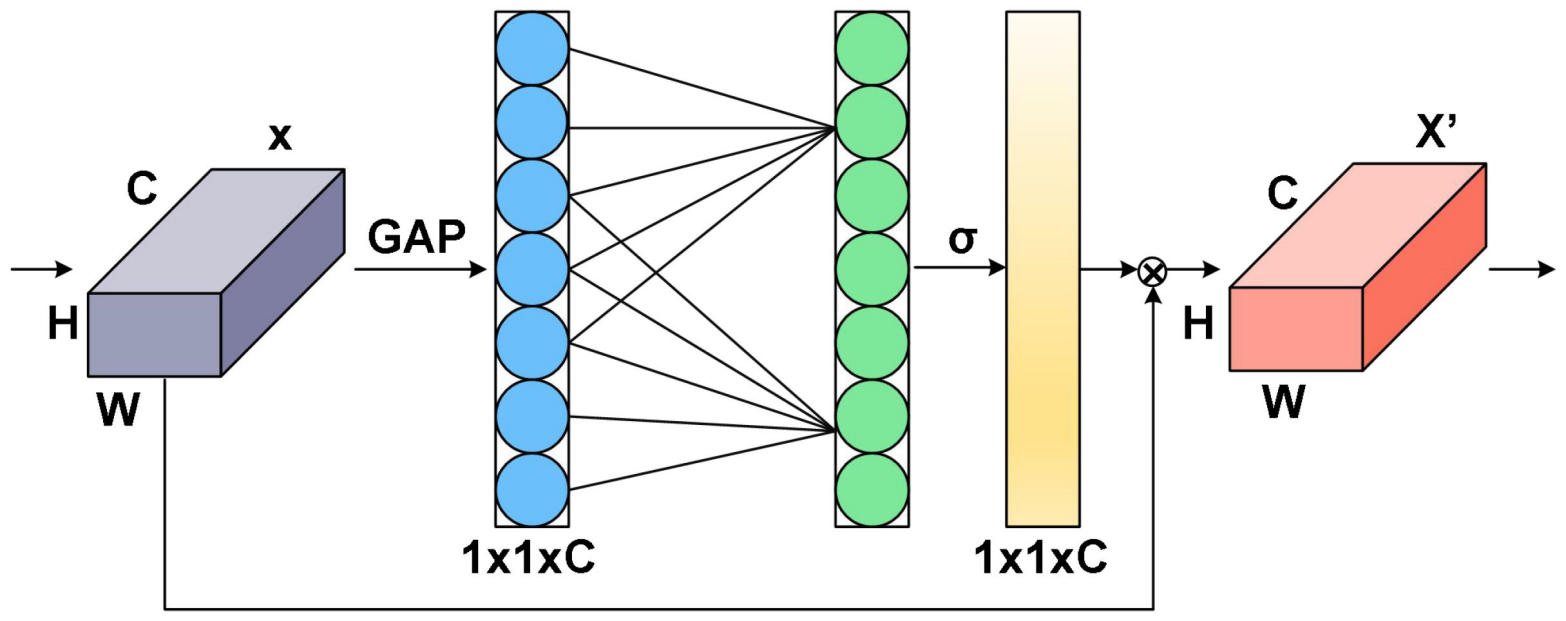
Schematic diagram of ECA mechanism.

ECA is an adaptive method for choosing the size of a one-dimensional convolution kernel, which improves upon the SE (Squeeze and Excitation) strategy by enabling local cross-channel interactions without dimensionality reduction (Qing and Liu, 2021; Huang et al., 2022; Zhao et al., 2022). To obtain unreduced features with a size of H×W×C , ECA first applies global average pooling (GAP) to the feature map with input size 1×1×C , as illustrated in **Figure 6**. Subsequently, ECA uses a one-dimensional convolution with a kernel size of k to recover the feature relationship of local k channels in the 1×1×C features and enable information interaction between channels. The input channel number C is used to adaptively determine the parameter k, as shown in equation (4),

ECA is an adaptive approach to selecting the size of a one-dimensional convolution kernel, which improves upon the SE method by allowing for local cross-channel interactions without reducing dimensionality. To obtain unreduced features with a size of H×W×C , ECA initially applies global average pooling (GAP) to the feature map with an input size of 1×1×C , as depicted in **Figure 6**. ECA then utilizes a one-dimensional convolution with a kernel size of k to restore the feature relationship of local k channels in the 1×1×C features and enable information interaction between channels. The input channel number C is employed to adaptively determine the parameter k, as shown in equation (4):


(4) $$k={\left|x\right|}_{odd}={\left|\frac{\log_{2}C+1}{2}\right|}_{odd}$$

where C represents the total number of input channels and ${\left|x\right|}_{odd}$ represents the odd number closest to x.

#### 2.2.4 Incorporation of adaptive feature fusion mechanism

**Figure 7:**
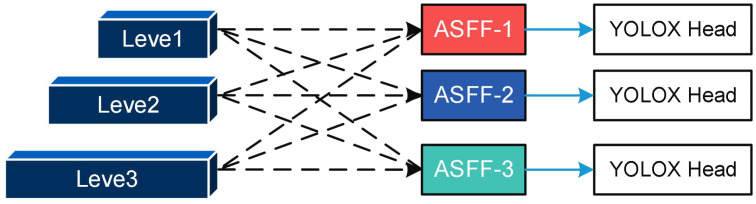
The schematic diagram of ASFF.

The original fusion method used by the YOLOX target detection network simply resizes the feature maps before adding them together, which does not fully exploit the features at different scales (Tang, 2022). In this study, we introduce an adaptive feature fusion approach to fully leverage the low-level contour, edge, color, and shape information, as well as the high-level semantic information of Papilionidae larva images. The structural diagram of the adaptive feature fusion method is depicted in **Figure 7**.

The YOLOX neck only outputs level, level2, and level3 feature maps. However, as shown in **Figure 7**, the output of the fused ASFF-3 module is obtained by multiplying the semantic properties of level, level2, and level3 by the weights of different layers, namely α, β, and γ, respectively. This approach enables the fully adaptive fusion of features from different levels, resulting in improved target detection performance.


(5) $$y_{ij}^{1}=\alpha_{ij}^{1}\times x_{ij}^{1\to 1}+\beta_{ij}^{1}\times x_{ij}^{2\to 1}+\gamma_{ij}^{1}\times x_{ij}^{3\to 1}$$

where, $\alpha_{ij}^{1}$ , $\beta_{ij}^{1}$ , $\gamma_{ij}^{1}$ are weights from different layers, $x_{ij}^{1\to 1}$ , $x_{ij}^{2\to 1}$ , $x_{ij}^{3\to 1}$ are outputs from different feature maps.

To combine the outputs of different levels in ASFF-3, it is critical to first compress level1 and level2 into the same number of channels using a 1×1 convolution kernel and then upsample them to match the dimension of level3. This is because the output of ASFF-3 is a fusion of three parts, and the resulting tensors after compression and upsampling are denoted as resize_level1 and resize_level2, respectively. Next, α, β and γ are computed by convolving resize_level1, resize_level2, and level3 with a 1×1 kernel. To ensure that α, β, and γ fall within the range of [0, 1], they are then normalized. The computation process is outlined in equation (6).


(6) $$\alpha_{ij}^{1}=\frac{e^{\alpha_{ij}^{1}}}{e^{\alpha_{ij}^{1}}+e^{\beta_{ij}^{1}}+e^{\gamma_{ij}^{1}}}$$

#### 2.2.5 Soft DIoU_nms to improve the detection performance of occluded insects

In the prediction stage, blindly removing prediction frames larger than the threshold in cases of dense overlapping of larvae may suppress the prediction frames for other Papilionidae larvae and prevent the detection of occluded overlapping pests. To address this issue, this article introduces the soft DIoU_nms, which modifies the intersection over union (IoU) calculation method in the non-maximum suppression (NMS) algorithm. The IoU computation process is outlined in equation (7).


(7) $$IoU=\frac{E\cap F}{E\cup F}$$

where *F* represents one of the remaining prediction boxes, and *E* represents the one with the highest current confidence score.

**Figure 8:**
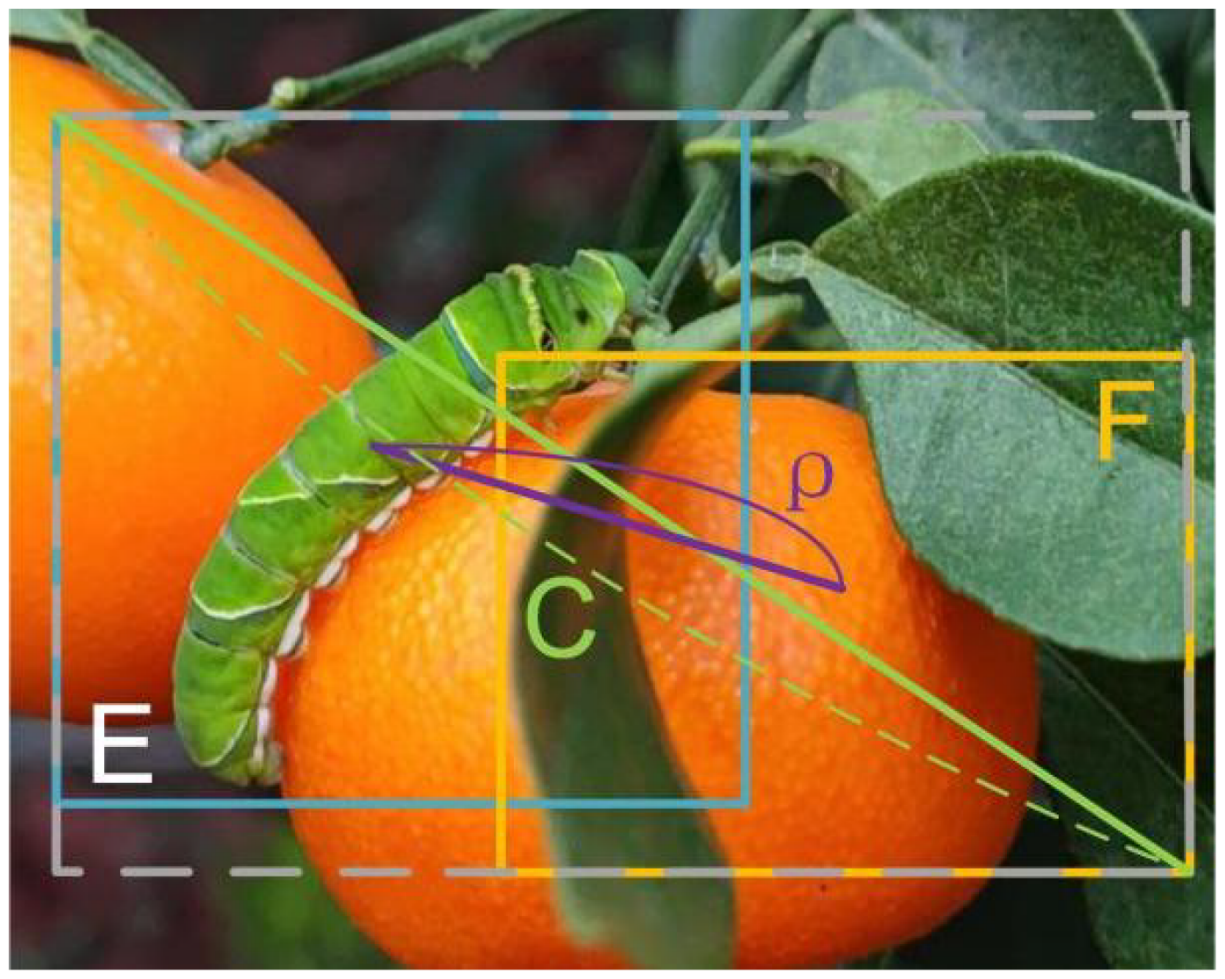
The schematic diagram of DIoU. ('E' represents the bounding box with the highest confidence, 'F' denotes other bounding boxes that do not have the highest confidence, and 'C' refers to the diagonal length of the minimum enclosing area comprising boxes 'E' and 'F').


In this work, DIoU is used instead of IoU. As shown in **Figure 8**, the blue rectangle E is the bounding box with the highest confidence, and the yellow rectangle F is one of the other bounding boxes. The DIoU computation process is outlined in equation (8).

(8) $$DIoU=IoU-\frac{\rho^{2}(b^{F},b^{E})}{c^{2}}$$

where $b^{F}$ is the center of box F, and $b^{E}$ is the center of box E. $\rho^{2}(b^{F},b^{E})$ represents the square of the distance between the center of frame E and frame F, and C represents the diagonal length of the minimum closure area between frame E and frame F.

The distances between bounding box centers are also considered in DIoU, which is used in this paper instead of IoU to improve accuracy and filter bounding boxes. When filtering additional redundant bounding boxes, soft DIoU_nms decreases their confidence rather than deleting all boxes above the threshold. The soft DIoU_nms computation process is outlined in equation (8).


(9) $$S_{i}=\left\{ \begin{array}{l} S_{i},DIoU(M,b_{i})<N_{t}\\ S_{i}e^{-\frac{DIoU{\left(M,b_{i}\right)}^{2}}{\sigma }},DIoU(M,b_{i})\geq N_{t} \end{array}\right.$$

where $S_{i}$ is the confidence score of the current prediction frame, M is the prediction frame with the highest confidence among all prediction boxes, $b_{i}$ represent the ith box in all compared prediction boxes in the current target, $N_{t}$ is the set threshold, generally 0.5, σ is the penalty item coefficient.

Soft DIoU_nms selects the prediction box with the highest score as the reference box, calculates the DIoU with the remaining prediction boxes within the current target, and retains the prediction box whose DIoU is below the set threshold. Instead of setting the boxes with a DIoU greater than the threshold to 0, their confidence score is gradually reduced. This approach allows some high-scoring boxes to be considered correct detection boxes in subsequent calculations. Thus, the use of soft DIoU_nms can significantly improve the detection performance of occluded overlapping IPPs, as demonstrated by equation (9).

#### 2.2.6 ASFL-YOLOX network design

**Figure 9:**
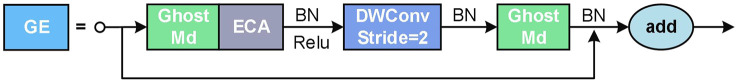
The GE neural network module.

**Figure 9** illustrates the Ghost ECA (GE) lightweight feature extraction module that integrates the Ghost Bottleneck and ECA mechanisms. The Ghost Bottleneck reduces computation and model parameters, while the ECA mechanism serves as a lightweight attention mechanism between the Ghost Bottleneck and Ghost module, improving the detection accuracy of the model for pests blocked by leaves and branches.

The TS activation function replaces the SiLU function of the detection head to further improve the performance of the Papilionidae larvae detection model. The TS activation function is outlined in equations (10) and (11).


(10) $$softplus(x)=\log\left(1+e^{x}\right)$$

(11) TS=tanh(x)·softplus(x)

**Figure 10:**
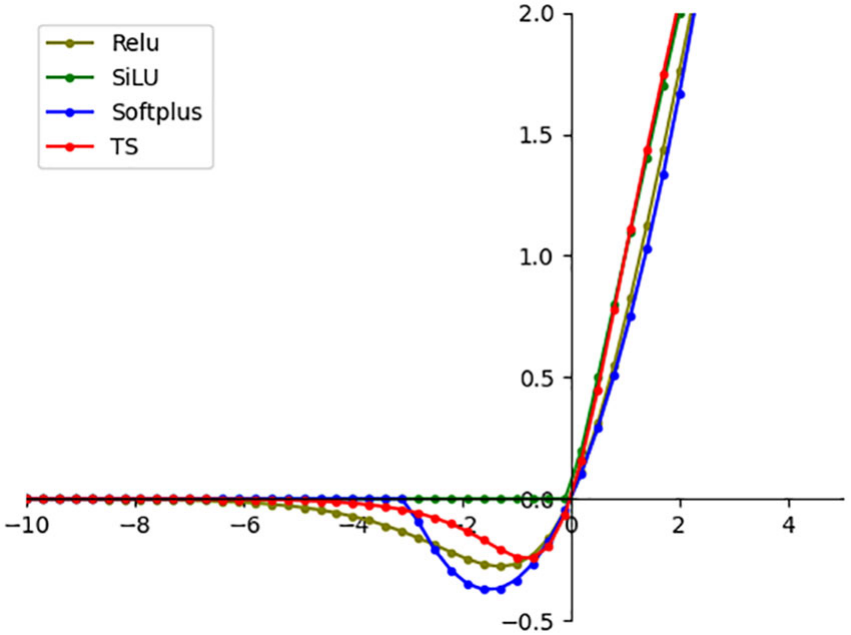
Activation function curve comparison diagram.

**Figure 10** illustrates the curves of the TS function and other activation functions. The TS function exhibits smoother characteristics compared to other functions, which is beneficial for enhancing the detection model’s performance.

**Figure 11:**
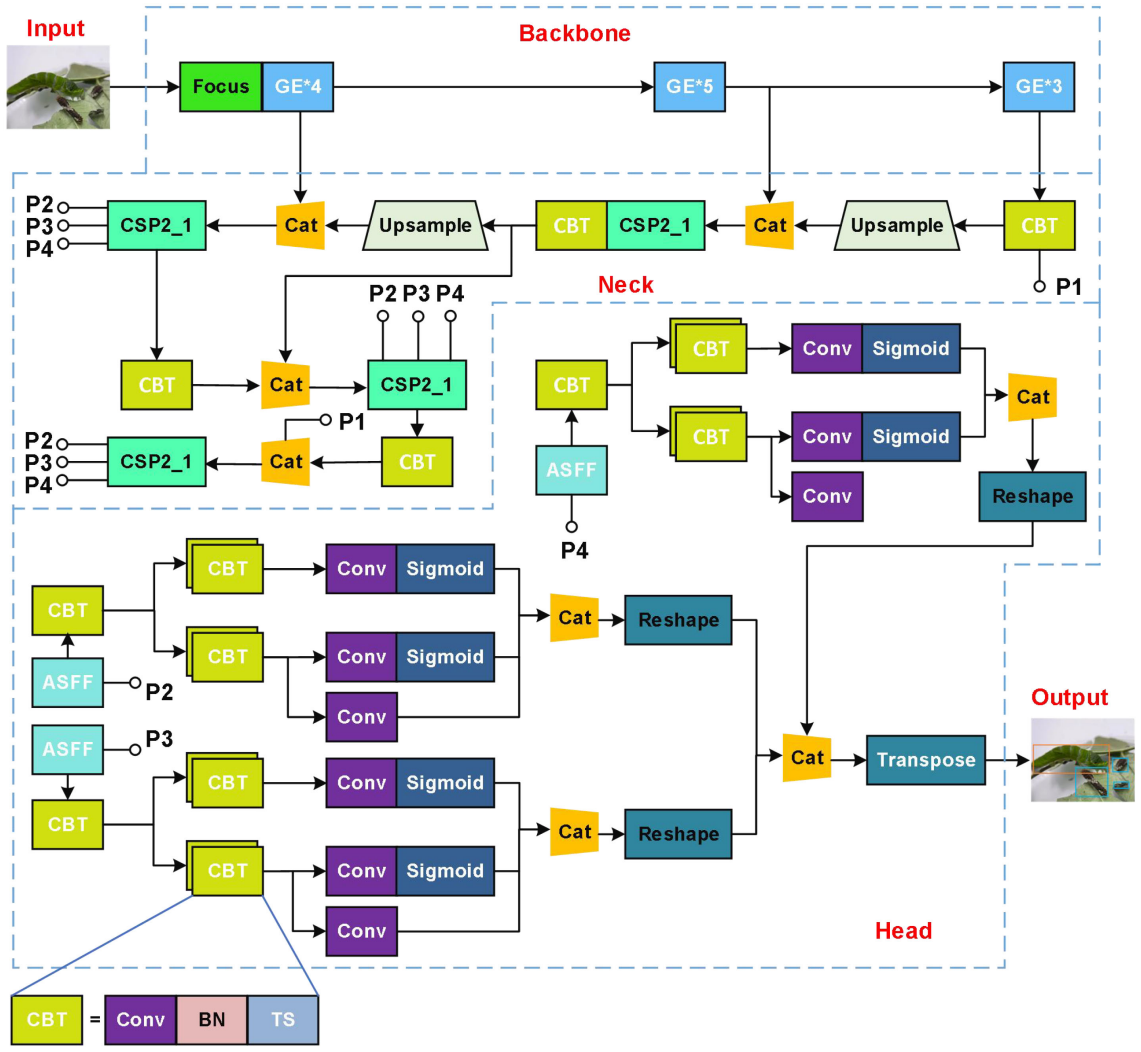
Structure of ASFL-TOLOX for IPPs detection.

In summary, we present a lightweight detection model, ASFL-YOLOX, for IPPs by integrating transfer learning, adaptive feature fusion, and attention guidance to enhance the network based on the high-performance detector YOLOX-S in this paper. **Figure 11** illustrates the network structure of our novel IPPs detection model.

**Figure 12:**
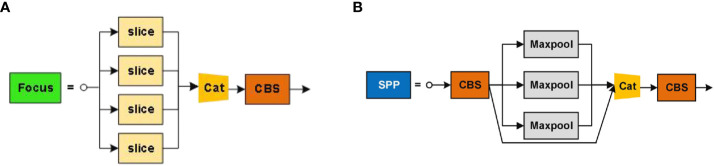
Structures of two key modules in ASFL-YOLOX: **(A)** Structure of Focus; **(B)** Structure of SPP.

The focus module structure, depicted in (**Figure 12A**), utilizes a slicing operation that samples the feature point information at intervals and stacks it on the channel, which is equivalent to dividing a high-resolution image into multiple low-resolution images. This operation avoids the loss of image information while downsampling the feature image. The spatial pyramid pooling (SPP**)** layer, shown in (**Figure 12B**), addresses the inconsistency in input image sizes by fusing multiple receptive fields using three different pooling operations.

#### 2.2.7 Introduction of structured pruning strategy to compress model

To improve the portability of ASFL-YOLOX, the best-performing pest detection model was first selected as the base model. Sparse training was then performed on the network model to prune unimportant channels, and fine-tuning was used to recover accuracy. The Batch Normalization (BN) layer was used to suppress the internal covariate shift, reducing the network model’s sensitivity to the initial parameter values and effectively improving the model’s convergence speed. The BN layer can be mathematically expressed as:

To improve the portability of ASFL-YOLOX, we first selected the best-performing pest detection model as the base model. Sparse training was then performed on the network model to prune unimportant channels, and fine-tuning was used to recover accuracy. The batch normalization (BN) layer was used to suppress internal covariate shifts, reducing the network model’s sensitivity to the initial parameter values and effectively improving the model’s convergence speed. The BN layer can be mathematically expressed in equation (12).


(12) $$z_{out\;}=\gamma \frac{z_{in\;}-\mu_{B}}{\sqrt{\sigma_{B}^{2}+\epsilon }}+\beta$$

where, $z_{in\;}$ and $z_{out\;}$ are the input and output data of the BN layer respectively, B is the current small batch; $\mu_{B}$ and $\sigma_{B}$ are the mean and standard deviation of the input data of B batches, γ and β are the scaling and translation parameters that can be learned during the training process, ϵ is a small amount that prevents the denominator from being zero.

The activation value of each channel is positively correlated with the learnable parameter γ, with channel-level scaling indicating that the parameter size directly affects the importance of the channel information. Therefore, the parameter γ is used as a quantitative index to measure the importance of the channel, also known as the scaling factor. Under normal conditions, the activation values output by the BN layer are normally distributed, with the majority of them not approaching zero. To facilitate sparse training and learning, the L1 regular constraint is introduced to reduce the value of the channel importance quantification index γ. The loss function is outlined in equation (13).


(13) $$L=L_{baseline\;}+\lambda \sum_{\gamma \in \Gamma }g(\gamma )$$

where $L_{baseline\;}$ is the loss function of the base model, $\sum_{\gamma \in \Gamma }g(\gamma )$ is the L1 regular constraint element, and g(γ)=│γ│ , λ is the penalty factor used to offset the loss element.

**Figure 13:**
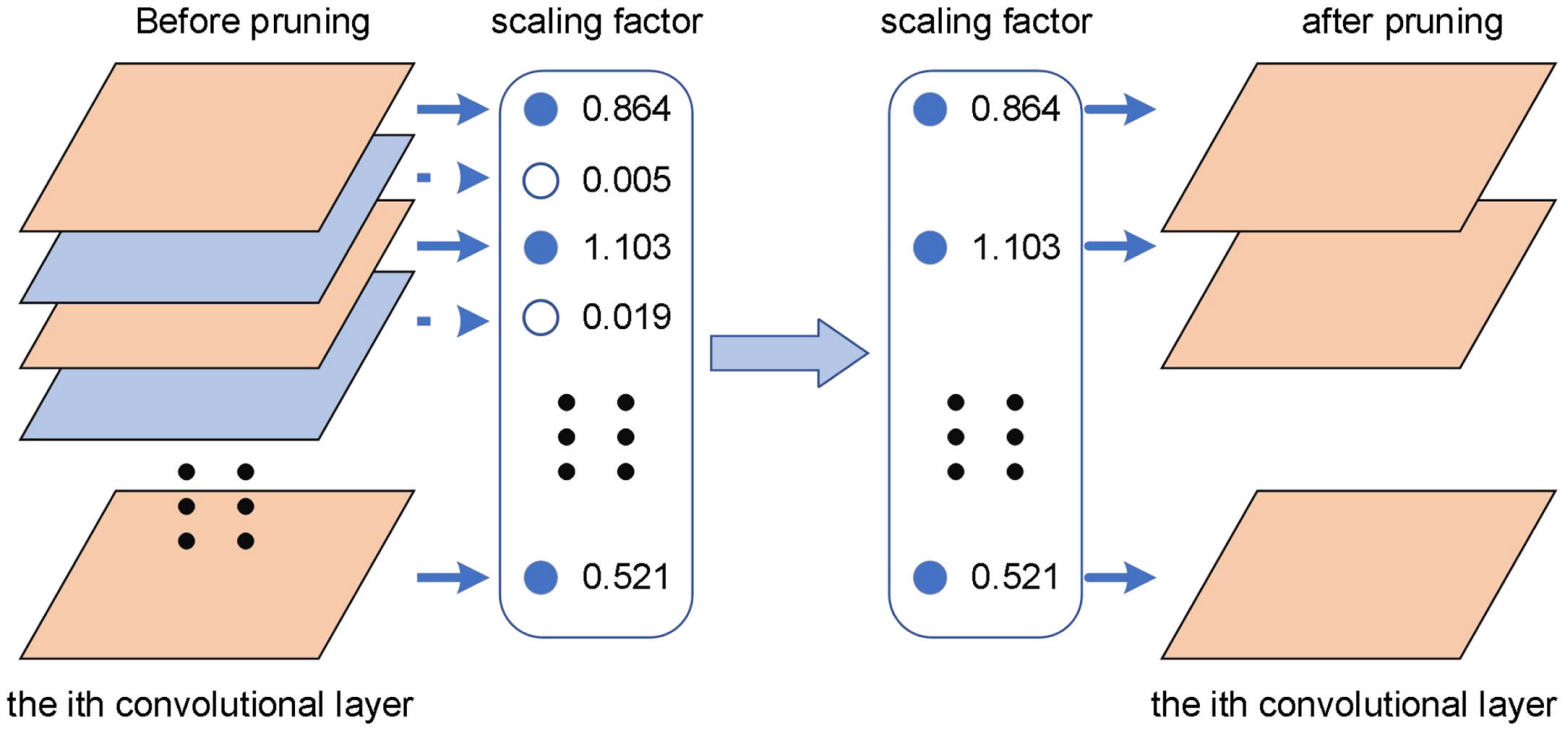
Schematic diagram of the model pruning process.

To obtain a highly sparse model with a slight loss of precision, sparse training requires adjusting the penalty factor and selecting an appropriate learning rate based on the weight distribution and average precision of the BN layer. **Figure 13** depicts the pruning process after sparse training of the model.

The scaling factor in the BN layer as a whole tends to zero, and the channel with the closest γ to zero is less important. Based on this, the scaling factors of all channels are sorted, and an appropriate pruning ratio is determined. The pruning ratio has an immediate impact on the model’s volume and accuracy. The greater the pruning ratio, the more channels are pruned, and the model becomes smaller, but the model’s accuracy suffers. As a result, after pruning the model, the accuracy is recovered through fine-tuning.

### 2.3 Evaluation indicators

To evaluate the detection performance of ASFL-YOLOX on IPPs, we introduce seven parameters and their calculations as equation (14)~(20).


(14) $$recall=\frac{TP}{TP+FN}$$

(15) $$precision=\frac{TP}{TP+FP}$$

(16) $$F1=\frac{2\times precision\times recall}{precision+recall}$$

(17) $$AP=\int_{0}^{1}P(R)dR$$

(18) $$FLOP\;\text{S}=2\times H\times W\left(C_{in}K^{2}+1\right)C_{out}$$

(19) $$Params=C_{\text{in }}\times K^{2}\times C_{\text{out }}$$

(20) $$FPS=\frac{N}{T}$$

where TP represents the number of true positive samples, FP represents the number of false positive samples, FN represents the number of false negative samples, and AP is the area enclosed by the Precision-Recall curve and the coordinate axis, with values ranging from 0 to 1, H and W represent the width and height of the input feature map, respectively, K represents the size of the convolution kernel, $C_{in}$ and $C_{out}$ indicate the input and output convolution kernel sizes, T indicates the total time used to detect all the images, N is the total number of images.

## 3 Experimental procedures and results analysis

### 3.1 Experiment configuration and hyperparameter selection

The experimental environment was built on top of the PyTorch deep learning framework, with GPU-accelerated processing. **Table 3** presents the hardware and software configuration of the experimental computer.

**Table 3:** The hardware and software configuration of the experimental computer.

hardware or software	model or version
CPU	IntelICoreIi9-10900K
GPU	NVIDIA Quadro RTX 5000
OS	Windows 10 enterprise 22H2
CUDA	CUDA10.0
CUDNN	CUDNN7.4.1
PyTorch	Pytorch_1.8.1

The ASFL-YOLOX network was trained using transfer learning, with the input image tensors of size (640, 640, 3). To allocate more resources to training the second half of the network, we first froze the backbone network’s pre-training weights and trained the network for 100 epochs. The following are the main hyperparameters for this process: batch size was set to 32, momentum factor was set to 0.93, the initial weight learning rate was set to 0.001, and decay coefficient was set to 0.0005. Subsequently, the network was unfrozen and trained for another 100 epochs. The following are the main hyperparameters for this process: epoch was set to 100, batch size was set to 16, impulse factor was set to 0.93, the initial learning rate of weight was set to 0.0001, and decay coefficient was set to 0.0005. Therefore, ASFL-YOLOX was trained for a total of 200 epochs. This training method has been proven to effectively avoid destroying backbone weights while also increasing training efficiency.

During the training process, the network’s learning rate was adjusted using the cosine annealing decay learning rate method, and the learning rate η can be expressed as shown in equation (21), where the smoothing label was set to 0.01. Cross mini-batch normalization (CmBN) regularization was used to update the network layer weights, and a weight file was stored in the training set at each epoch.


(21) $$\eta_{t}=\frac{1}{2}\left(1+\cos\left(\frac{t\pi }{T}\right)\right)\eta$$

where t indicates the batch size, and T indicates the epoch number.

The original model’s weight parameters were used as initialization weights in the sparse training process, with a penalty factor of 0.001, a learning rate of 0.0001, a batch size of 16, and 100 iterations. To avoid a significant loss of accuracy, we used a pruning rate of 65% based on the distribution of scaling factors. After pruning, we fine-tuned the model to improve its accuracy. The warm-up learning rate optimization method was used in this process, with a small learning rate in the early stages of training to avoid overfitting. The learning rate was gradually reduced as the number of iterations increased to speed up the model’s convergence. Finally, when the model’s training was stable, a smaller learning rate was used to avoid destroying the model’s stability.

**Figure 14:**
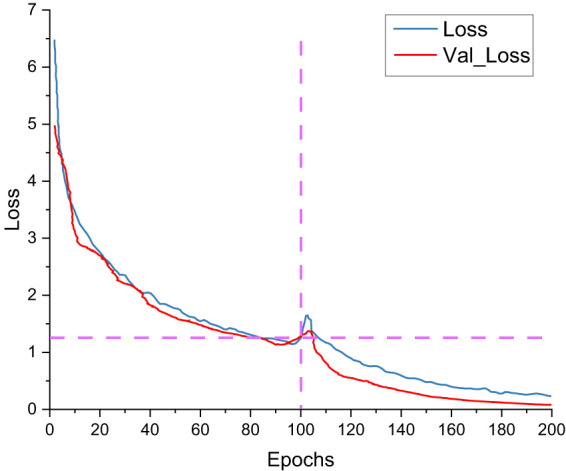
ASFL-YOLOX loss curve.

**Figure 14** shows the training set loss and validation set loss of the ASFL-YOLOX model during training. The graph shows that the ASFL-YOLOX model’s training set loss and validation set loss change trends are essentially the same. The loss curve gradually stabilizes as the training times increase, the model gradually converges, and the loss decreases very rapidly for the first 20 training times. The loss decreases dramatically when the backbone is unfrozen in the 100th training session. Like the previous 100 training times, the model gradually approaches and converges on the optimal point until the training reaches 190 times.

### 3.2 Ablation experiment

ASFL-YOLOX enhances the YOLOX-x network with four evaluation parameters used in the ablation experiment: mAP, Params, FLOPs, and FPS. The experimental results are displayed in **Table 4**.

**Table 4:** Ablation experiment results.

methods	GhostNet	ECA	ASFF	TS	soft DIoU_nms	pruning	mAP (%)	Params (M)	FLOPs (G)	FPS
YOLOX-x	×	×	×	×	×	×	96.64	99.10	141.90	22
improvement 1	√	×	×	×	×	×	87.47	45.16	83.75	43
improvement 2	√	√	×	×	×	×	90.98	51.32	94.66	33
improvement 3	√	√	√	×	×	×	93.59	55.88	101.91	27
improvement 4	√	√	√	√	×	×	94.85	55.67	100.89	27
improvement 5	√	√	√	√	√	×	96.52	55.67	100.89	27
our method	√	√	√	√	√	√	95.76	10.93	30.87	66

(The symbol "×" indicates that a certain innovation point was not adopted in the ablation experiment, while "√" indicates that a certain innovation point has been adopted in the ablation experiment).

**Table 4** presents that replacing the YOLOX-x backbone network with GhostNet decreases its mAP, Params, and FLOPs by approximately 9.17%, 53.94%, and 58.15G, respectively while increasing its FPS by approximately 21. Incorporating the ECA mechanism increases the network model’s mAP, Params, and FLOPs by about 3.51%, 6.16%, and 10.19G, respectively, while the FPS decreases by about 10. Following the addition of the ASFF module based on the above, the network model’s mAP, Params, and FLOPs increase by 2.61%, 4.56%, and 7.25G, respectively, while the FPS decreases by about 5.

After replacing the SiLU activation function with the TS function, the network model’s mAP increases by approximately 1.26%, while its Params and FLOPs decrease by 0.21% and 1.02G, respectively, and its FPS remains unchanged. The network model’s mAP further increases by about 1.67% after optimizing the maximum value suppression method, while its parameters, FLOPs, and FPS remain unchanged. Finally, using the pruning strategy to compress the network model reduces its mAP, Params, FLOPs, and FPS by 0.76%, 88.17%, 111.03G, and 50.00%, respectively.

**Figure 15:**
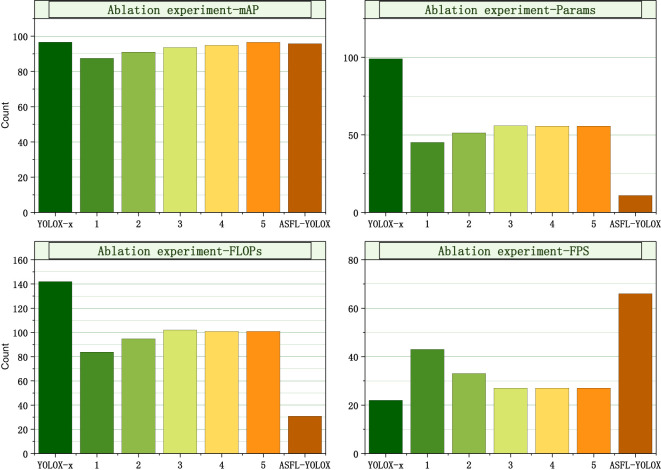
Assessing model performance via ablation: comparison of four indicators.

**Figure 15** shows that the mAP value of the ASFL-YOLOX model is slightly lower than the YOLOX-x model’s 96.64%. However, other indicators of the ASFL-YOLOX model (such as Params, FLOPs, and FPS) have significantly improved. Specifically, the ASFL-YOLOX model utilizes fewer parameters and computational resources while maintaining a high mAP value and fast inference speed.

### 3.3 Comparison of different attention mechanisms

Through ablation experiments, it is evident that incorporating an attention mechanism into YOLOX-x can significantly improve the model’s detection accuracy. To further prove that adding ECA to the YOLOX-x network is more suitable for IPPs detection, an attention mechanism comparison experiment is designed to compare the detection performance of the network model by adding three mainstream attention mechanisms of SE, CBAM, and ECA, respectively. **Table 5** displays the results.

**Table 5:** Results of the comparison experiment of different attention mechanisms.

Model	mAP (%)	Params (M)	FLOPs (G)	FPS
YOLOX_x	96.64	99.10	141.90	22
YOLOX-x+SE	96.97	105.77	152.16	15
YOLOX-x+CBAM	97.31	110.64	155.23	10
YOLOX-x+ECA	98.14	105.26	152.81	12

**Figure 16:**
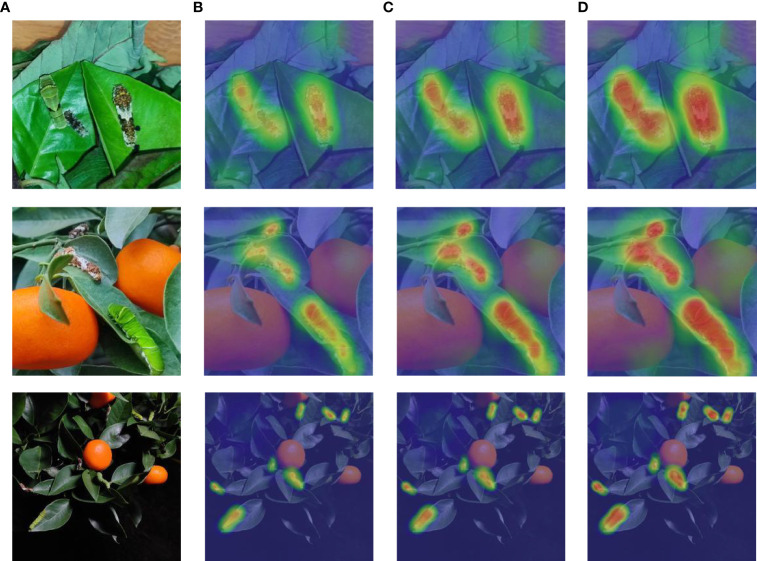
Heat map of model detection results with different attention mechanisms: **(A)** original image; **(B)** YOLOX-x+SE; **(C)** YOLOX-x+CBAM; **(D)** YOLOX-x+ECA.

The experiment results show that all three attention mechanisms can improve YOLOX-x detection accuracy, but at varying degrees of computational cost. Compared to YOLOX-x, the mAP, Params, and FLOPs of the “YOLOX-x+SE” model increased by 0.33%, 6.67M, and 10.26G, respectively, but its FPS decreased significantly. The “YOLOX-x+CBAM” model’s mAP, Params, and FLOPs increased by 0.67%, 11.54M, and 13.33G, respectively. However, the computational cost of this model is relatively high, and it is not suitable for real-time detection. When compared to YOLOX-x, the mAP, Params, and FLOPs of the “YOLOX-x+ECA” model increased by 1.5%, 6.16M, and 10.91G, respectively. It is evident that adding ECA to the YOLOX-x model achieves a better balance of performance and computational cost than adding SE and CBAM. And **Figure 16** shows the heat map of the detection results of the YOLOX-x model under the three attention mechanisms generated by the Grad-CAM++ algorithm.

The heat map clearly shows the area that the network model pays attention to, and the redder the part of the image, the more attention the network model pays to that area. When comparing the generated activation heat maps, it is clear that ECA focuses more clearly on the body area of the IPPs larvae and can locate the larvae more accurately.

### 3.4 Comparison of classic target detection models

The ASFL-YOLOX model was compared to other classical models to validate the superiority of the proposed method in this paper, i.e., the longitudinal comparison experiment. Firstly, the YOLOX-s, YOLOX-m, YOLOX-l, YOLOX-x, YOLOX-Darknet53, YOLOX-Nano, and YOLOX-Tiny models were chosen as longitudinal comparison models, and their performance on the test set was recorded. The results are shown in **Table 6**, which indicates that the ASFL-YOLOX model performed well in all indicators for detecting young larvae. Its precision, recall, AP, and F1 values were all maintained at higher thresholds while being time-saving. The mAP value of ASFL-YOLOX was 95.76%, while YOLOX-nano and YOLOX-tiny only achieved 48.73% and 53.36%, respectively. The model with the highest mAP value is YOLOX-x, but it requires a large number of parameters and FLOPs, resulting in a low FPS. The overall performance of the ASFL-YOLOX model in detecting old larvae remains the best.

**Table 6:** Longitudinal comparison results.

		Ours	YOLOX-s	YOLOX-m	YOLOX-l	YOLOX-x	YOLOX-Darknet53	YOLOX-Nano	YOLOX-Tiny
Young	P (%)	95.82	86.33	91.35	94.89	96.89	93.55	47.79	54.11
	R (%)	94.74	83.98	88.33	93.97	93.97	91.97	42.66	50.93
	AP (%)	95.91	84.21	89.48	94.67	96.67	92.52	46.39	51.24
	F1 (%)	95.28	85.14	89.81	94.43	95.41	92.75	45.08	52.47
Old	P (%)	96.94	87.46	92.66	95.81	97.01	94.67	50.03	58.96
	R (%)	95.92	85.11	89.53	95.44	96.44	92.90	49.68	54.66
	AP (%)	95.61	85.80	90.79	95.04	96.61	94.18	51.07	55.47
	F1 (%)	96.43	86.27	91.07	95.62	96.72	93.78	49.85	56.73
Comprehensive index	mAP (%)	95.76	85.01	90.14	94.86	96.64	93.35	48.73	53.36
	Params(M)	10.93	9.00	25.30	54.20	99.10	63.70	0.91	5.06
	FLOPs(G)	30.87	26.75	76.38	95.76	141.90	105.38	1.98	8.45
	FPS	66	70	43	31	22	26	128	101

Next, Faster R-CNN, SSD, YOLOv7, and other models were chosen as latitude comparison models, trained with the same training parameters and datasets, and their performance on the test set was compared. **Table 7** displays the results, which show that while the AP, Precision, and F1 values of ASFL-YOLOX are not the highest, it is light and fast in inference. ASFL YOLOX’s mAP is 11.84% higher than YOLOv3, and its AP value is 6.92% higher than YOLOv4. In terms of detection speed, Faster-RCNN has an FPS of 19, which is significantly lower than SSD and YOLO series networks, indicating its limitations in real-time detection. The average detection accuracy of YOLOv7-x is 96.98%, but its detection speed is 37 FPS, which is slower than ASFL-YOLOX. ASFL-YOLOX has an FPS of 66, which is 29 higher than the FPS of YOLOv7-x, and its mAP is about 10 percentage points higher than that of YOLOv7-tiny. Although ASFL-average YOLOX’s accuracy is slightly lower than that of YOLOv7-x, the latter’s higher complexity results in slower recognition speed. Therefore, the ASFL-YOLOX series outperforms the YOLOv7 series in terms of overall performance and model size.

**Table 7:** Latitude comparison results.

		Ours	Faster R-CNN	SSD	CenterNet	EfficientDet	FCOS	YOLOv3	YOLOv4	YOLOv5_x	YOLOv7_x	YOLOv7_Tiny
Young	P (%)	95.82	82.15	84.43	80.92	60.87	75.61	86.78	88.69	93.89	97.02	86.92
	R (%)	94.74	80.39	83.02	77.23	59.20	71.09	83.99	87.17	92.11	95.77	83.06
	AP (%)	95.91	81.07	83.45	78.15	61.59	73.34	85.36	88.44	93.05	96.89	84.96
	F1 (%)	95.28	81.26	83.72	79.03	60.02	73.28	85.36	87.92	92.99	96.39	84.95
Old	P (%)	96.94	82.71	83.08	81.11	63.77	80.19	86.98	90.03	95.44	97.78	87.03
	R (%)	95.92	83.66	80.17	79.04	60.95	77.16	86.11	91.12	94.87	95.99	84.59
	AP (%)	95.61	83.32	81.91	79.88	62.22	81.23	85.87	90.68	95.02	97.06	85.41
	F1 (%)	96.43	83.18	81.60	80.06	62.33	78.65	86.54	90.57	95.15	96.88	85.79
Comprehensive index	mAP (%)	95.76	82.20	82.68	79.02	61.91	77.29	85.62	89.56	94.04	96.98	85.19
	Params (M)	10.93	136.70	23.61	32.67	3.87	31.84	61.52	63.93	86.70	71.30	6.01
	FLOPs (G)	30.87	171.51	31.39	22.15	2.29	47.58	116.40	101.57	157.83	92.69	10.13
	FPS	66	19	52	57	113	46	29	33	20	37	118

**Figure 17:**
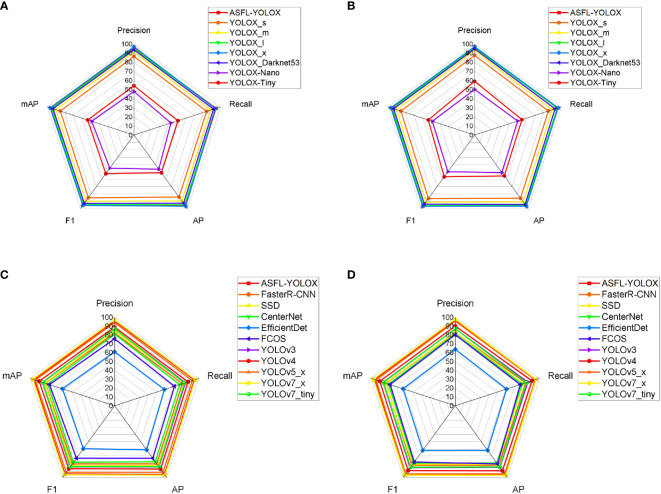
Radar chart of longitudinal and latitude comparison: **(A)** Longitudinal comparison-Young; **(B)** Longitudinal comparison-Old; **(C)** Latitude comparison-Young; **(D)** Latitude comparison-Old.

**Figure 18:**
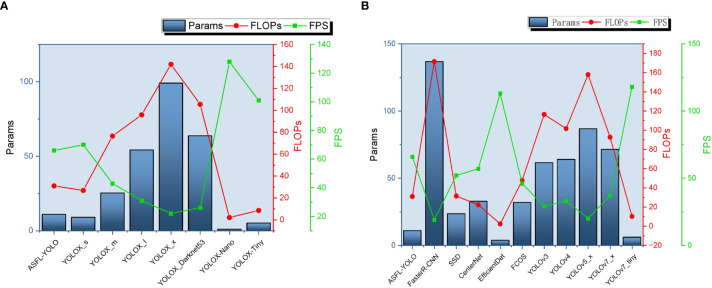
The model complexity is compared in terms of latitude and longitude, where panel **(A)** presents the results of the longitude comparison and panel **(B)** displays the results of the latitude comparison.

A comprehensive analysis of **Tables 6**, **7** enables the creation of performance and complexity comparison charts for each model, as depicted in **Figures 17**, **18**, respectively.

**Figure 17** illustrates the vertical and horizontal comparison model performance radar chart, showing that the differences in the ASFL-YOLOX model indicators are small between young and old targets, indicating that the model has good robustness. Precision, Recall, AP, F1, mAP, and other indicators are compared, indicating that ASFL-YOLOX can improve overall detection performance.

**Figure 18** is a comparison chart of vertical and horizontal model complexity. It compares Params, FLOPs, and FPS indicators, showing that the ASFL-YOLOX model has smaller Params and FLOPs than other models, but its FPS is comparable to the YOLOX-S and YOLOX-Nano models, indicating that ASFL-YOLOX has advantages in model size and computational efficiency. In conclusion, ASFL-YOLOX can improve overall detection performance while retaining a small model size and high computational efficiency.

### 3.5 Real scene detection experiments

#### 3.5.1 Detection under different degrees of occlusion

In unstructured orchards, branches, and leaves often obstruct or overlap insects, leading to varying degrees of occlusion. This paper uses the degree of occlusion as the control variable and the test sets A, B, and A+B as experimental data to compare the detection results of the ASFL-YOLOX model and two lightweight models, YOLOX-s and YOLOv5-s, as shown in **Table 8**.

**Table 8:** Comparison of detection results with different occlusion degrees.

model	test set	R (%)	P (%)	F1 (%)	mAP (%)
YOLOv5-s	A	85.67	90.12	87.84	83.24
	B	79.24	87.85	83.32	77.13
	A+B	82.16	88.11	85.03	81.22
YOLOX-s	A	86.61	93.25	89.81	91.28
	B	80.12	90.10	84.82	84.69
	A+B	84.55	86.90	85.70	85.01
ASFL-YOLOX	A	96.54	96.82	96.68	96.83
	B	92.75	94.41	93.57	93.14
	A+B	95.33	96.38	95.85	95.76

The experimental results indicate that the ASFL-YOLOX model outperforms the YOLOv5-s and YOLOX-s models in terms of performance indicators (precision, recall, F1, and mAP) on datasets A, B, and A+B especially for severely occluded data. **Table 8** shows that as the degree of occlusion increases, the detection performance of each model decreases, but the performance decrease of ASFL-YOLOX is smaller. The other two models perform relatively poorly on datasets B and A+B, possibly due to the increased difficulty of target detection with increasing occlusion degree. The ASFL-YOLOX model is optimized for occlusion to better identify occluded targets.

**Figure 19:**
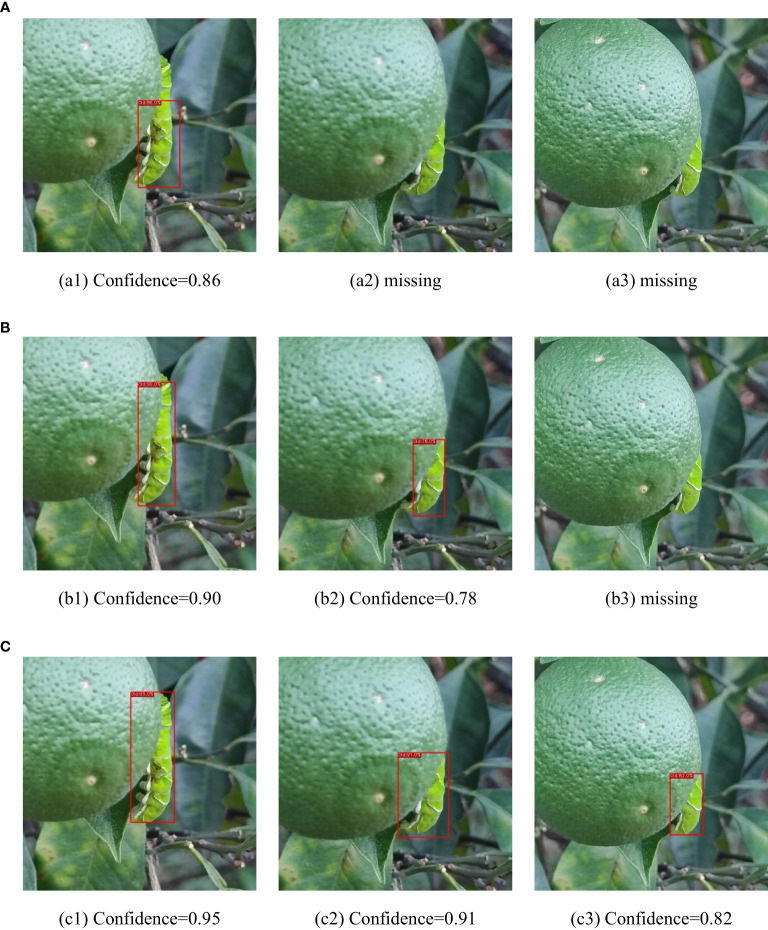
The detection results of various models under different levels of insect body obstruction are as follows: **(A)** shows the detection results of YOLOv5, **(B)** presents the detection results of YOLOX, and **(C)** illustrates the detection results of ASFL-YOLOX.

**Figure 19** presents the measured results of the three models on different occlusion test sets. For the light occlusion test set, all three models successfully detect the pests in the image, but ASFL-YOLOX exhibits higher confidence. ASFL-YOLOX has a significant confidence advantage in the heavy occlusion test set. Both YOLOX and YOLOv5 exhibit varying degrees of missed detection, with low confidence scores. In summary, ASFL-YOLOX has a higher detection rate and a confidence score for heavily occluded pests, reducing the missed detection rate of occluded pests.

#### 3.5.2 Detection under different lighting angles

We conducted tests under front lighting, side lighting, and backlighting conditions to evaluate the ASFL-YOLOX model’s robustness under different lighting angles, and the results are presented in **Tables 9**, **10**.

**Table 9:** Detection results of different lighting angles in the unstructured orchard.

lighting angle	model	R (%)	P (%)	F1 (%)	mAP (%)
front lighting	YOLOv5_s	87.71	92.65	90.11	85.72
	YOLOX_s	88.97	94.41	91.61	93.37
	ASFL-YOLOX	95.51	97.93	96.70	98.07
side lighting	YOLOv5_s	82.77	89.21	85.87	81.09
	YOLOX_s	84.56	91.11	87.71	90.09
	ASFL-YOLOX	94.88	95.98	95.43	96.60
backlighting	YOLOv5_s	77.23	80.12	78.65	75.17
	YOLOX_s	80.61	87.11	83.73	84.68
	ASFL-YOLOX	91.41	93.04	92.22	93.71

**Table 10:** Detection results of different lighting angles in unstructured orchards.

model	front lighting	side lighting	backlighting
YOLOv5_s	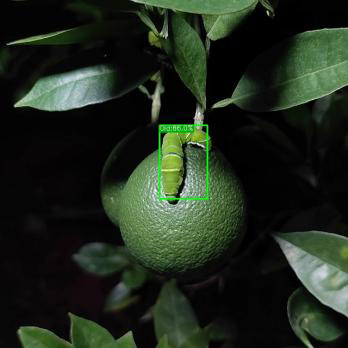	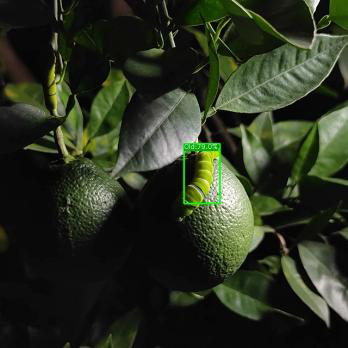	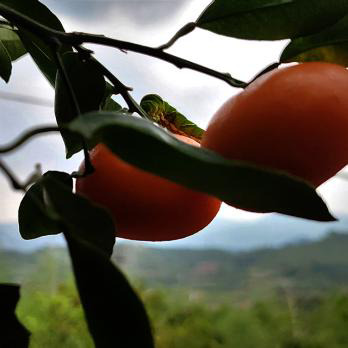
	(a1) confidence=0.86	(a2) confidence=0.79	(a3) missing
YOLOX_s	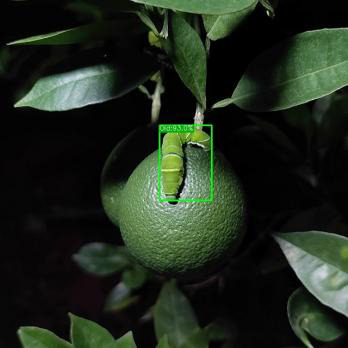	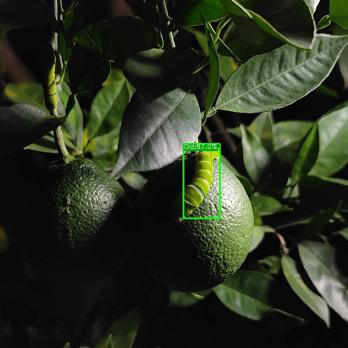	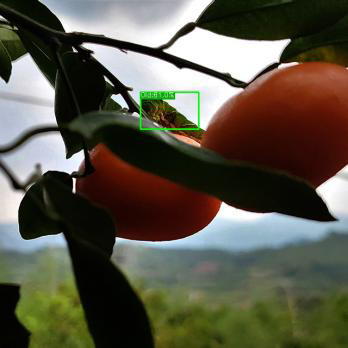
	(b1) confidence=0.93	(b2) confidence=0.87	(b3) confidence=0.81
Ours	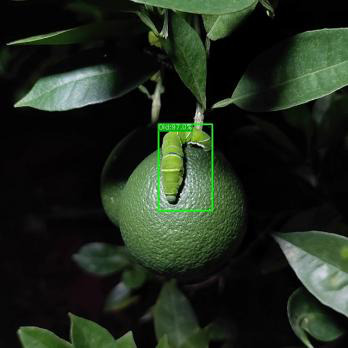	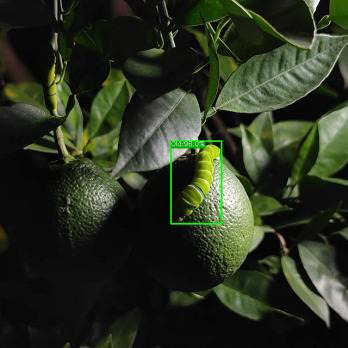	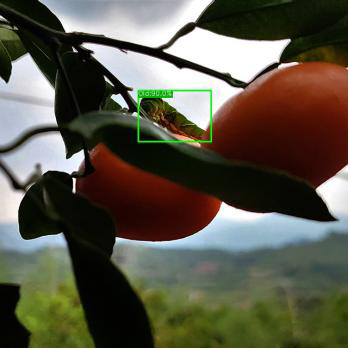
	(c1) confidence=0.97	(c2) confidence=0.96	(c3) confidence=0.90

The precision, recall, F1, and mAP values of the ASFL-YOLOX model are significantly better than those of the YOLOv5-s and YOLOX-s models under the three different lighting conditions, as shown in **Tables 9**, **10**. For instance, when tested with front lighting data, the ASFL-YOLOX model achieved precision, recall, F1, and mAP values of 97.93%, 95.51%, 96.70, and 98.07%, respectively, outperforming the other two models. The same trend was observed under side-lit and backlit conditions. Thus, the ASFL-YOLOX model demonstrates superior detection performance and adaptability to varying lighting conditions. **Table 10** illustrates that under backlighting conditions, the image color and texture features are lost due to insufficient light, leading to a high-contrast image. At this point, the YOLOv5 model experiences missed detection, while the ASFL-YOLOX model still has the highest confidence.

## 4 Discussions

This article uses GhostNet (Xu et al., 2023) to replace the backbone network of YOLOX, which not only stabilizes the average accuracy but also greatly reduces the number of network parameters. **Table 4** verifies that GhostNet reduces the model Params by 54.43%, FLOPs by 40.98%, and increases FPS by 21 frames/s; the structured pruning strategy reduces the model Params by 80.37%, FLOPs by 69.40%, and increases FPS by 39 frames/s; ASFL-YOLOX reduces the model parameters by 88.97% compared to YOLOX-x, and FLOPs are compressed from 141.90G to 30.87G. In **Table 7**, the inference speed of ASFL-YOLOX is 3.5 times that of the Faster R-CNN series models, indicating that the Faster R-CNN series models have greater limitations in terms of inference speed, and ASFL-YOLOX has obvious advantages. **Table 4** verifies that after using ECA, the model mAP is increased by 3.51%. **Figure 16** further proves that ECA is more suitable for application in the detection task of Papilionidae pests. Zha et al. (2021) proposed a lightweight pest detection model YOLOv4_MF, which used Adaptive Spatial Feature Fusion (ASFF) as part of the BA module to improve the detection accuracy of the model. We draws on Zha’ s ideas and uses ASFF to connect YOLOX’s decoupled head. **Table 4** verifies that ASFF can increase the model mAP by 2.61%. We innovatively proposes a TS activation function to replace the SiLU activation function. **Figure 10** confirms that the TS activation function has smoother characteristics, that is, it has better generalization ability and effective optimization ability. In addition, this article is based on the Distance-IoU (DIoU) loss function and combines the advantages of traditional NMS methods and DIoU_NMS methods to use soft DIoU_NMS algorithms to optimize the screening of prediction boxes. In **Figure 8**, soft DIoU_NMS can more accurately evaluate the overlap degree of candidate boxes and adjust according to different situations, which further improves the accuracy and performance of the target detection model. **Table 4** points out that TS and soft DIoU_NM respectively increase the model mAP by 1.26% and 1.67%. Through multiple measures to improve model accuracy, ASFL-YOLOXs mAP reached 95.75%, so this article’s improvement measures can solve the problem of accuracy decline caused by model lightweighting, although ASFL-YOLOX’s mAP is slightly lower than YOLOX-x, But ASFL-YOLOX has better overall performance.

Due to the complexity of the orchard environment and the varying sizes of insects at different growth stages, the model in this study still has some issues with false detections when detecting pests in complex backgrounds, and the recognition performance for “young” insects is inferior to that of “old” insects. In response to these shortcomings, future expansion of data sets can further improve the generalization and robustness of models. In summary, We proposes a neural network model ASFL-YOLOX that can accurately and quickly identify Papilionidae pests.

## 5 Conclusions

In this paper, we propose a lightweight real-time detection method, called ASFL-YOLOX, for the larvae of IPPs. To achieve network lightweight, we use GhostNet as the backbone network. We replace the CBS module in the Head with the CBT module, which is composed of the TS activation function, to further reduce the model’s memory occupancy. We also introduce the ECA mechanism at critical positions in the network to suppress interference from complex backgrounds. Moreover, we use the soft DIoU_nms algorithm to enhance the recognition capability of overlapping or occluded pests.

Comparative experimental results of various models show that the ASFL-YOLOX network’s detection performance is significantly better than classical target detection models such as Faster-RCNN, SSD, and YOLOv7 when detecting IPPs in unstructured orchards. Especially in cases of severe insect occlusion, compared with the traditional YOLOX network, the ASFL-YOLOX network has a higher average detection accuracy, a more lightweight model size, and faster inference speed. This method considers the model size, inference speed, and detection accuracy, making it more suitable for deployment on embedded devices and mobile terminals. Furthermore, this method can be applied to other agricultural products and positively promotes the development of agricultural spraying robots.

## Data availability statement

The original contributions presented in the study are included in the article/supplementary material. Further inquiries can be directed to the corresponding authors.

## Author contributions

LX and XS conceived and designed the study. ZT and YH performed the experiments and collected the data. NY and WM analyzed the data and performed the statistical tests. CZ, HC, and TZ developed the software and tools for data processing. PH, ZW, and YW contributed to the interpretation of the results and provided critical feedback. XS and LX wrote the first draft of the manuscript. ZZ, JD, and KZ revised the manuscript. YZ supervised the study and approved the final version of the manuscript. All authors read and agreed to be accountable for all aspects of the work.
